# Characterization of resistance genes and replicon typing in Carbapenem-resistant *Klebsiella pneumoniae* strains

**DOI:** 10.1186/s12941-024-00672-9

**Published:** 2024-02-24

**Authors:** Esin Karaman, Ayşegül Çopur Çiçek, Vicdan Şemen, Fatih Şaban Beriş

**Affiliations:** 1 Faculty of Arts&Sciences, Department of Biology, Recep Tayyip Erdogan University, Rize, Turkey; 2 Faculty of Medicine, Department of Medical Microbiology, İstanbul Medipol University, İstanbul, Turkey; 3 Department of Microbiology, Sakarya Yenikent State Hospital, Sakarya, Turkey

**Keywords:** *K. pneumoniae*, Carbapenem resistance, Antibiotic susceptibility, Antibiotic resistance genes, Replicon typing

## Abstract

**Objective:**

In our study, *K. pneumoniae* strains (non-susceptible to carbapenem) (*n* = 60) were obtained from various clinical samples from Rize State Hospital between 2015 and 2017 and it is aimed to identify antibiotic resistance genes and replicon typing.

**Methods:**

Antibiotic susceptibility tests of the strains were performed with Kirby-Bauer disk diffusion test and the Vitek-2 automated system (BioMerieux, France). Antibiotic resistance genes and replicon typing was characterized by PCR method.

**Results:**

It was determined that *K. pneumaniae* isolates were mostly isolated from the samples of the intensive care unit. All of the *K. pneumoniae* strains examined in this study were found to be ampicillin/sulbactam and ertapenem resistant but colistin susceptible. Amoxacillin/clavulonic acid resistance was detected at 98.14% of strains. The *bla*_OXA-48_ gene was mostly detected in isolates. The most common type of plasmid was *I1* and 3 different plasmid types were found in five different strains together.

**Conclusion:**

This study also shows that the distribution of NDM-1 and OXA-48 carbapenemases has increased since the first co-display in Türkiye and that *IncHI1* is the first record in our country. This study provides an overview of the major plasmid families occurring in multiple antibiotic-resistant strains of *K. pneumoniae*. To our knowledge, this study represents the first report of *IncHI1* record in Türkiye.

## Background

The increase in antimicrobial resistance is a concern for human and animal health all over the world. Globally, 700.000 people die each year from infections caused by antimicrobial resistance [1, 2]. *Klebsiella pneumoniae* is a gram-negative opportunistic pathogen that causes healthcare-associated infections in hospitalized and immunocompromised individuals [1]. In neonates, it is one of the leading causes of sepsis in patients with hematological malignancies [3, 4]. It may colonize the gastrointestinal tract, skin, nose and throat of healthy individuals [5]. It has recently acquired additional genetic features, the number of severe infections due to *K. pneumoniae* increased and treatment efficacy decreased due to the emergence of hypervirulent (HV) or antibiotic-resistant *K. pneumoniae* strains [6]. *K. pneumoniae* has a high resistance to many antibiotic groups, such as beta-lactam antibiotics, fluoroquinolones, and aminoglycosides [7]. Antimicrobial resistance is commonly associated with the spread of infectious plasmids and the acquisition of resistance genes that normally occur by horizontal gene transfer and can also carry virulence factors [8]. The increase in the clinical use of carbapenems has led to the emergence of carbapenem-resistant *K. pneumoniae* worldwide in recent years. Carbapenem-resistant *K. pneumoniae* is mainly associated with metallo-β-lactamases, such as *K. pneumoniae* carbapenemase (KPC), oxacillinase-48 (OXA-48), and New Delhi metallo-β-lactamase (NDM), imipenemase (IMP), and Verona integron‒encoded metallo-β-lactamase (VIM). Although the prevalence of these plasmid-mediated carbapenemases in the world varies geographically, it has been reported many times that OXA-48 producers are the source of nosocomial outbreaks since their first detection in Türkiye [9].

The emergence of carbapenem-resistant *K. pneumoniae* strains with increased virulence has made it difficult to distinguish between these strains and hypervirulent strains. Therefore, the increasing association between carbapenem resistance and hypervirulence in *K. pneumoniae* is alarming [10]. Horizontal gene transfer via plasmids plays an important role in increasing bacterial antibiotic resistance (AMR). The identification of plasmid properties and their relationship to different bacterial hosts provides information about the contribution of plasmids to AMR delivery. Molecular identification of these plasmid and progeny genotypes provides an understanding of the difference between the expression of AMR genes by plasmids and the expression of these genes by bacteria [11]. Plasmid typing can provide insight into the epidemiology of resistance plasmids [12]. In particular, PCR-based replicon typing (PBRT) is frequently used for typing plasmids [11]. In Enterobacterales, including *K. pneumoniae*, there are 28 plasmid types that can be distinguished by PBRT [13].

Our study aimed to identify non-carbapenem-susceptible *K. pneumoniae* strains sent from various clinics in Rize State Hospital (RSH) in 2015–2017, which were found to have developed resistance in recent years and to determine their antibiotic resistance profiles and responsible genes for resistance. Plasmid typing was performed by examining replicon types. Since RSH functions not only as a local but also as a regional hospital. The antibiotic resistance profile of *K. pneumoniae* in various clinical samples, the detection of the resistance genes, and the examination of the spread of these genes have potential effects on infection control, public health, and rational antibiotic use.

## Materials and methods

### Obtaining and identifying isolates

Sixty *K. pneumoniae* strains isolated from patients treated at Rize State Hospital between 2015 and 2017 were included in the study. Samples were stocked at -20 °C and − 80 °C to contain 20% glycerol. Identification of strains were identified and verified by conventional methods and the Vitek-2 automated (BioMerieux, France).

### Antimicrobial susceptibility test

Antibiotic susceptibility tests were determined by Kirby-Bauer disk diffusion test and the Vitek-2 automated system (BioMerieux, France). This information was taken retrospectively from the hospital information management system. For antibiotic susceptibility testing: amikacin, amoxicillin-clavulanic acid, ampicillin/sulbactam, cefepime, cefotaxime, ciprofloxacin, colistin, ertapenem, imipenem, meropenem, piperacillin/tazobactam, tigecycline, gentamicin discs were studied at 18–24 °C for 18 h of plaques. Inhibition zone diameters around the antibiotic discs were measured following the incubation of antibiotics, and the results were evaluated according to the Clinical and Laboratory Standards Institute (CLSI) criteria [3].

### Stocking of *K. pneumoniae* isolates and total DNA isolation

*K. pneumoniae* isolates were grown in 4 mL of antibiotic-free Luria-Bertani (LB) medium and grown in a shaking incubator at 37 °C overnight. By taking 800 µL of bacterial suspensions, 20% glycerol stocks were prepared. 1000 µL of the remaining bacterial suspensions were centrifuged at 14,000 rpm for 5 min. The pellets were dissolved in 1000 µL of distilled water and boiled for 10 min. After centrifugation at 14,000 rpm for 10 min, 500 µL of the supernatant was used as DNA source [4].

### Screening of antibiotic resistance genes in *K. pneumoniae* strains by PCR

Antibiotic resistance genes (*bla*_TEM_, *bla*_SHV_, *bla*_CTX-M1_, *bla*_CTX-M2_, *bla*_GES_, *bla*_VEB_, *bla*_PER_, *bla*_KPC_, *bla*_IMP_, *bla*_VIM_, *bla*_NDM_, *bla*_OXA-51_, *bla*_OXA-58_, *bla*_OXA-23_, *bla*_OXA-40_, and *bla*_OXA-48_) of *K. pneumoniae* strains isolated from hospital infections were characterized by PCR method. Primers in Table 1 were used to search for genes responsible for antibiotic resistance by PCR. 5 µL of genomic DNA, 1 µL of each primer, 5 µL of reaction buffer, 3 µL of 25 mM MgCl_2_, 2.5 µL of 4 mM dNTP and 0.2 µL of *Taq* Polymerase were added to a reaction mixture and the final volume was made up to 50 µL with distilled water. PCR results will be analyzed on 1% agarose containing 0.5 mg/L ethidium bromide and then visualized under UV light. As the PCR conditions, the conditions specified in the relevant references in Table 1 were used. Amplicons of the size specified in the reference studies were accepted as positive.

**Table 1:** Primers sequences and their futures for detection of *β*-lactamase encoding genes

Genes	Primers (5’→3’)	Amplicon size (bp)	Tm (°C)	References
*bla*_TEM_	F: AGTATTCAACATTTYCGTGT R: TAATCAGTGAGGCACCTATCTC	847	56	[5]
*bla*_SHV_	F: ATGCGTTATATTCGCCTGTG R: TTAGCGTTGCCAGTGCTC	843	55	
*bla*_CTX−M1_	F: GCGTGATACCACTTCACCTC R: TGAAGTAAGTGACCAGAATC	260		
*bla*_CTX−M2_	F: TGATACCACCACGCCGCTC R: TATTGCATCAGAAACCGTGGG	341		
*bla*_GES_	F: ATGCGCTTCATTCACGCAC R: CTATTTGTCCGTGCTCAGGA	863	56	[4]
*bla*_VEB_	F: ATTTCCCGATGCAAAGCGT R: TTATTCCGGAAGTCCCTGT	542	55	
*bla*_PER−2_	F: ATGAATGTCATCACAAAATG R: TCAATCCGGACTCACT	927	50	
*bla*_IMP_	F: CATGGTTTGGTGGTTCTTGT R: ATAATTTGGCGGACTTTGGC	488	56	
*bla*_VIM_	F: ATTGGTCTATTTGACCGCGTC R: TGCTACTCAACGACTGAGCG	780	58	
*bla*_NDM−1_	F: GAGATTGCCGAGCGACTTG R: CGAATGTCTGGCAGCACACTT	497	54	
*bla*_OXA−23_	F: GATCGGATTGGAGAACCAGA R: ATTTCTGACCGCATTTCCAT	501	52	
*bla*_OXA−24_	F: GGTTAGTTGGCCCCCTTAAA R: AGTTGAGCGAAAAGGGGATT	246		
*bla*_OXA−51_	F: TAATGCTTTGATCGGCCTTG R: TGGATTGCACTTCATCTTGG	353		
*bla*_OXA−58_	F: AAGTAT TGGGGCTTGTGCTG R: CCCCTCTGCGCTCTACATAC	599		
*bla*_KPC_	F: CGTTCTTGTCTCTCATGGCC R: CCTCGCTGTGCTTGTCATCC	796	52	[6]
*bla*_OXA−48_	F: TTGGTGGCATCGATTATCGG R: GAGCACTTCTTTTGTGATGGC	743	57	

### Determination of replicon types

Plasmid analysis in *K. pneumoniae* strains was investigated by PCR method. Specific primers for HI1, HI2, I1, X, L/M, N, FIA, FIB, W, Y, P, FIC, A/C, T, FIIs, FrepB, and K/B replicons by Carattoli [7]. Replicon typing was performed with (Table 2). Primers, target DNA sequence and PCR fragment size to be obtained are as in Table 2. PCR; 2.5 µL of 10X Buffer, 1.5 µL of 25 mM MgCl_2_, 1.25 µL of 4 mM dNTPs, 1 µL of each primers (10 pmol), 0.2 µL of 5 U/µL *Taq* DNA pol was prepared by completing 5 µL of template DNA, and the final volume to 25 µL sterile deionized water. As reaction cycling conditions using the primers shown in Table 2 were 94 °C for 5 min (1 cycle), 30 cycles of 1 min at 94 °C, 30 s at 60 °C and 1 min at 72 °C, and the final synthesis was used at 72 °C for 5 min. The PCR products and 1 kbp DNA ladder (Thermo, USA) were run on a 1% agarose gel containing 0.5 mg/L ethidium bromide. Then, it was decided whether the expected amplicon sizes were obtained by visualizing them under UV light.

**Table 2:** List of primers used in plasmid typing studies [7]

Replicon Type	Primers (5’$\rightarrow$3’)	Target Region	EMBL Accesion Number	Amplicon size (bp)
HI1	F: GGAGCGATGGATTACTTCAGTAC	*par*A-*par*B	AF250878	471
	R: TGCCGTTTCACCTCGTGAGTA			
HI2	F: TTTCTCCTGAGTCACCTGTTAACAC	Iterons	BX664015	644
	R: GGCTCACTACCGTTGTCATCCT			
I1	F: CGAAAGCCGGACGGCAGAA	RNAI	M20413	139
	R: TCGTCGTTCCGCCAAGTTCGT			
X	F: AACCTTAGAGGCTATTTAAGTTGCTGAT	*ori*g	Y00768	376
	R: TGAGAGTCAATTTTTATCTCATGTTTTAGC			
L/M	F: GGATGAAAACTATCAGCATCTGAAG	*rep*A, B, C	U27345	785
	R: CTGCAGGGGCGATTCTTTAGG			
N	F: GTCTAACGAGCTTACCGAAG	*rep*A	NC_003292	559
	R: GTTTCAACTCTGCCAAGTTC			
FIA	F: CCATGCTGGTTCTAGAGAAGGTG	Iterons	J01724	462
	R: GTATATCCTTACTGGCTTCCGCAG			
FIB	F: GGAGTTCTGACACACGATTTTCTG	*rep*A	M26308	702
	R: CTCCCGTCGCTTCAGGGCATT			
W	F: CCTAAGAACAACAAAGCCCCCG	*rep*A	U12441	242
	R: GGTGCGCGGCATAGAACCGT			
Y	F: AATTCAAACAACACTGTGCAGCCTG	*rep*A	K02380	765
	R: GCGAGAATGGACGATTACAAAACTTT			
P	F: CTATGGCCCTGCAAACGCGCCAGAAA	Iterons	M20134	534
	R: TCACGCGCCAGGGCGCAGCC			
FIC	F: GTGAACTGGCAGATGAGGAAGG	*rep*A2	AH003523	262
	R: TTCTCCTCGTCGCCAAACTAGAT			
A/C	F: GAGAACCAAAGACAAAGACCTGGA	*rep*A	X73674	465
	R: ACGACAAACCTGAATTGCCTCCTT			
T	F: TTGGCCTGTTTGTGCCTAAACCAT	*rep*A	K00053	750
	R: CGTTGATTACACTTAGCTTTGGAC			
FIIs	F: CTGTCGTAAGCTGATGGC	*rep*A	AE006471	270
	R: CTCTGCCACAAACTTCAGC			
F_repB_	F: TGATCGTTTAAGGAATTTTG	RNAI/*rep*A	AY234375	270
	R: GAAGATCAGTCACACCATCC			
K/B	F: GCGGTCCGGAAAGCCAGAAAAC	RNAI	M93063	160
	R: TCTTTCACGAGCCCGCCAAA			
B/O	R: TCTGCGTTCCGCCAAGTTCGA	RNAI	M28718	159

## Results

Most of the *K. pneumaniae* isolates were from the intensive care unit (*n* = 24). Detailed strain information is given in Table 3.

**Table 3:** Medical units from which *K. pneumoniae* isolates are obtained

Clinics	Number of isolates (%)
Nephrology	8 (13.3%)
Surgical	6 (10%)
Intensive care	24 (40%)
Orthopedics	1 (1.6%)
Cardiology	1 (1.6%)
Pediatrics	2 (3.3%)
Ear nose throat	1 (1.6%)
Gynecology	2 (3.3%)
İnfectious diseases	2 (3.3%)
Urgent	2 (3.3%)
Hematology	5 (8.3%)
Gastroenterology	1 (1.6%)
Neurology	3 (5%)
Urology	2 (1.6%)
TOTAL	60 (100%)

The resistance rates of *K. pneumoniae* strains to amikacin, amoxycillin-clavulonic acid, ampicillin, cefepime, cefotaxime, ciprofloxacin, colistin, ertapenem, imipenem, meropenem, piperacillin, tigecycline, and gentamicin were 1.7%, 98.4%, 100%, 65%, 71.7%, 0%, 100%, 76.7%, 85.04%, 96.7%, 15.1%, 38.4%, respectively. Accordingly, it was observed that the antibiotic with the highest resistance rate was ampicillin and ertapenem (100%), and the antibiotic with the lowest resistance rate was colistin (0%). In particular, carbapenem resistance was found to be significantly higher in strains. In our study, it was observed that *K. pneumoniae* strains showed resistance to all antibiotics except colistin, and all strains were susceptible to colistin (Table 4).

**Table 4:** Antibiotic susceptibility rates (*n* = 60)

Antibiotics	Resistant	Mid sensitive	Sensitive
Amikacin	1.7	13.3	85.0
Amoxycillin-Cavulonic acid	98.4	1.6	0.0
Ampicillin/Sulbactam	100.0	0.0	0.0
Cefepim	65.0	0.0	35.0
Cefotaxime	86.4	1.6	11.6
Ciprofloxacin	71.7	3.3	25.0
Colistin	0.0	0.0	100.0
Ertapenem	100.0	0.0	0.0
Imipenem	76.7	8.3	15.0
Meropenem	85.04	3.3	11.6
Piperacillin/Tazobactam	96.7	3.3	0.0
Tigecycline	15.1	31.6	53.3
Gentamicin	38.4	0.0	61.6

The presence of resistance genes was investigated by polymerase chain reaction (PCR) and at least one or more β-lactamase genes were detected in each sample (Table 5). In addition, more than one resistance gene association was detected in some strains. The rates of resistance genes alone or in combination are as in Tables 6 and 7. The most common *bla*_OXA-48_ gene was detected in isolates, and it was detected in fifty-eight (96.6%) of the isolates.

**Table 5:** PCR results of *β*-Lactamase resistance genes (*n* = 60)

Samples No.	NDM	GES	TEM	SHV	CTX-M-1	CTX-M-2	IMP	OXA-48
1			**+**	**+**		**+**		**+**
2			**+**	**+**				**+**
3			**+**	**+**	**+**			**+**
4			**+**	**+**	**+**			**+**
5			**+**	**+**	**+**			**+**
6			**+**	**+**	**+**			**+**
7			**+**	**+**	**+**			**+**
8			**+**	**+**	**+**			**+**
9			**+**	**+**	**+**			**+**
10			**+**	**+**	**+**			**+**
11			**+**	**+**				
12			**+**	**+**	**+**			**+**
13			**+**	-	**+**			**+**
14			**+**	**+**	**+**			**+**
15			**+**	**+**	**+**			**+**
16				**+**				**+**
17			**+**	**+**	**+**	+		**+**
18				**+**				**+**
19				**+**				**+**
20				**+**				**+**
21				**+**				**+**
22			**+**	**+**	**+**			**+**
23			**+**	**+**	**+**			**+**
24			**+**	**+**				**+**
25			**+**	**+**	**+**			**+**
26			**+**	**+**	**+**			**+**
27				**+**	**+**			**+**
28				**+**			**+**	
29		**+**	**+**	**+**	**+**			**+**
30			**+**	**+**				**+**
31			**+**	**+**			**+**	**+**
32			**+**	**+**			**+**	**+**
33			**+**	**+**	**+**			**+**
34			**+**	**+**				**+**
35			**+**	**+**				**+**
36			**+**	**+**	**+**		**+**	**+**
37			**+**	**+**	**+**			**+**
38			**+**	**+**	**+**			**+**
39			**+**	**+**		**+**		**+**
40			**+**	**+**		**+**		**+**
41				**+**			**+**	**+**
42			**+**	**+**	**+**			**+**
43							**+**	**+**
44			**+**	**+**	**+**	**+**		**+**
45			**+**	**+**	**+**			**+**
46	**+**		**+**	**+**	**+**	**+**		**+**
47				**+**	**+**	**+**		**+**
48				**+**				**+**
49			**+**	**+**	**+**			**+**
50			**+**	**+**	**+**		**+**	**+**
51	**+**		**+**	**+**				**+**
52	**+**		**+**	**+**	**+**	**+**		**+**
53			**+**	**+**	**+**			**+**
54			**+**	**+**	**+**	**+**		**+**
55			**+**	**+**	**+**			**+**
56								**+**
57				**+**	**+**	**+**		**+**
58			**+**		**+**			**+**
59	**+**		**+**		**+**		**+**	**+**
60			**+**	**+**	**+**	**+**		**+**

Note: Only those with resistance gene are marked as “+”. Samples without resistance gene were not labeled

**Table 6:** Distribution and gene associations of extended spectrum beta lactamase (ESBL) genes detected in *K. pneumoniae* isolates (*n* = 60)

ESBL genes	Positive strain number (n, %)
NDM	4 (6.6%)
GES	1 (1.6%)
TEM	47 (78.3%)
SHV	55 (91%)
CTX-M1	38 (63.3%)
CTX-M2	11 (18.3%)
IMP	8 (13.3%)
OXA-48	58 (96.6%)

**Table 7:** Gene associations of ESBL genes in strains (*n* = 60)

Gene associations	Number of cases (%)
TEM + SHV + CTX-M2 + OXA-48	3 (5.08%)
TEM + SHV + OXA-48	5 (8.4%)
TEM + SHV + CTX-M1 + OXA-48	23(38.9%)
TEM + SHV	1 (1.69%)
TEM + CTX-M1 + OXA-48	2 (3.2%)
SHV + OXA-48	6 (10.1%)
SHV + CTX-M1 + OXA-48	1 (1.69%)
SHV + IMP	1 (1.69%)
GES + TEM + SHV + CTX-M1 + OXA-48	1 (1.69%)
TEM + SHV + IMP + OXA-48	2 (3.38%)
TEM + SHV + CTX-M1 + IMP + OXA-48	2 (3.38%)
TEM + SHV + CTX-M1 + CTX-M2 + OXA-48	4 (6.7%)
SHV + IMP + OXA-48	1 (1.69%)
IMP + OXA-48	1 (1.69%)
NDM + TEM + SHV + CTX-M1 + CTX-M2 + OXA-48	2 (3.38%)
NDM + TEM + SHV + OXA-48	1 (1.69%)
SHV + CTX-M1 + CTX-M2 + OXA-48	2 (3.38%)
NDM + TEM + CTX-M1 + IMP + OXA-48	1 (1.69%)

*I1* plasmid type was found most frequently among the strains (*n* = 20, 33.3%). The *HI2*, *K*, *W*, *FIC*, *A/T*, *T*, *FIIs*, *K/B* replicon types could not be detected in any of the strains (Table 8). When evaluated in terms of plasmid associations, association was detected in 1 or two strains in 2 and 3 combinations (Table 9). *I1* + *FIB* + *Y* and *L*/*M* + *Y* plasmid associations were mostly found in 2 strains (6.25%). The most common combination of plasmid types was determined as *IncI1* + *IncFIB* + *IncY*, and *IncL/M* + *IncY*.

**Table 8:** Plasmid detected strains and their detected plasmid types

Samples No.	HI1	I1	L/M	N	FIA	FIB	Y	*P*	FrepB
3		+				+	+		
4		+				+			
5						+	+		
6							+		
7							+		
8							+		
9		+							
10		+							
11		+							
12		+							
13		+				+	+		
14						+	+		
15						+			
16		+				+		+	
17		+							
18		+	+		+				
21		+	+	+					
22		+					+		
23		+							
24		+							
26		+							
28		+							
29		+							
30		+							
32		+	+						
33	+								
34		+							
35			+				+		
36									+
37			+						
38							+		
50			+				+		

Note: The strain for which no replicon type was identified is not shown in the table. Only those with replicon were marked. HI1 (*n* = 1), I1 (*n* = 20), L/M (*n* = 6), N (*n* = 1), FIA (*n* = 1), FIB (*n* = 7), Y (*n* = 11), P (*n* = 1), FrepB (*n* = 1)

**Table 9:** Plasmids co-occurrence combinations and their rates

Plasmids types associations	Number of samples and their percentages (n, %)
I_1_ + FIB + Y	2 (6.25%)
I_1_ + FIB	1 (3.12%)
I_1_ + FIB + P	1 (3.12%)
I_1_ + L/M + FIA	1 (3.12%)
I_1_ + L/M + N	1 (3.12%)
I_1_ + Y	1 (3.12%)
I_1_ + L/M	1 (3.12%)
L/M + Y	2 (6.25%)
FIB + Y	1 (3.12%)

* Percentages in the number of cases were taken according to the number of strains (*n* = 32) with the plasmid detected

## Conclusion

The emergence of antibiotic-resistant infections is recognized as a serious problem for human health worldwide. The widespread use and misuse of antibiotics has led to a decrease in the effectiveness of conventional antimicrobial therapy and appropriate antibiotic selection [8]. In this study, isolation from urine in eighteen (30%) isolates and from blood in eleven (18.3%) isolates show that infections are most related to urinary system and bloodstream infections. According to the 2020 report of the Central Asian and European Surveillance of Antimicrobial Resistance (CAESAR) study, which also includes our country, the rate of carbapenem resistance in *K. pneumoniae* strains is 43% [10]. In this study, carbapenem resistance was found to be 87.2% in 60 *K. pneumoniae* strains, much higher than the Asian and European averages. All the *K. pneumoniae* strains examined in our study were found to be ampicillin/sulbactam and ertapenem resistant but colistin susceptible. Amoxacillin/clavulonic acid resistance was detected at 98.14% of strains. This also demonstrates the effects of commonly used antibiotics. The rates of strains carrying *bla*_GES_, *bla*_NDM_, *bla*_TEM_, *bla*_SHV_, *bla*_CTX-M1_, *bla*_CTX-M2_, *bla*_IMP_, and *bla*_OXA-48_ type resistance genes in their isolates were determined. The most common type was *bla*_OXA-48_ with 96.6%, followed by *bla*_SHV_ with 91.6%, *bla*_TEM_ with 78.3%, *bla*_CTX-M-1_ with 63.3%, *bla*_CTX-M-2_ with 18.3%, *bla*_IMP_ with 13.3%, followed 6.6% by *bla*_NDM_, and 1.6% by *bla*_GES_.

After the *bla*_OXA-48_ gene was first detected in *K. pneumoniae* in 2001, nosocomial outbreaks of *bla*_OXA-48_ positive *K. pneumoniae* have occurred within five years [11]. In the study by Ece et al. [12], all *K. pneumoniae* isolates were carbapenem resistant; all contained *bla*_OXA-48_ and all were sensitive to both gentamicin and colistin. In our study, 96.6% of carbapenem-resistant *K. pneumonia*e isolates contained *bla*_OXA-48_, and all were found to be susceptible to colistin and 61% to gentamicin. The reason for this high rate is that Turkey is the epicenter for *bla*_OXA-48_.

The *bla*_SHV1_ gene, which is the precursor of SHV-type β-lactamases, is most common in *K. pneumoniae* worldwide. Oksuz et al. in Türkiye, 92.9% of the *bla*_SHV_ and 52.2% of the *bla*_TEM_ gene was found in *K. pneumoniae* strains [14]. Similarly, in our study, *bla*_SHV_ was detected with a rate of 91.6% but *bla*_TEM_ was found in 78.3%. From the point of view of *bla*_CTX-M-1_ and *bla*_CTX-M-2_, they found 62-87.7% and 0-100% [15, 16]. In our study, *bla*_CTX-M-1_ was detected in 63.3% and *bla*_CTX-M-2_ in 18.3%. These results are within average for the presence of these genes. In terms of the presence of *bla*_NDM_ and *bla*_IMP_ genes, it was found to be 6.6% and 13.3% lower in carbapenem-resistant *K. pneumoniae* strains investigated compared to previous studies [13, 17]. In our study, *bla*_VIM_, *bla*_PER_, *bla*_VEB_, *bla*_KPC_, *bla*_OXA-23-24-51-58_ gene positive isolates were not detected in any of the 60 isolates. Similarly, *bla*_PER_ and *bla*_VEB_ type β-lactamases were not detected in any strain by Bektas et al. in our country [18]. In a study conducted by Sağıroğlu et al. [13]. in Türkiye, they found 2% of *bla*_KPC_ positivity. In our study, however, no *bla*_KPC_ resistant isolate was detected. This situation gives us concern that the *bla*_KPC_ resistance gene has not become widespread yet, but that *bla*_KPC_ resistance will increase in the future. It is thought that the knowledge gained by monitoring the resistance, conducting the necessary studies to prevent the development of resistance, systematically evaluating the data of our country, choosing the antimicrobial treatment correctly and appropriately, preventing unnecessary antibiotic use, ensuring the success of the treatment, and contributing to the country’s economy because of these are thought to be valuable.

Plasmid profiles vary among the *K. pneumoniae* isolates we studied. Plasmid profiles were found in a total of 26 isolates, and 12 different plasmid profiles containing one or more plasmids were found in these isolates. The most common *IncI1* resistance plasmid profile was found in 76.9% (20/26). It is followed by *IncY* (30.7%;8/26), *IncFIB* (26.9%;7/26), *IncL/M* (11.5%;3/26), *IncHI1* (3.8%;1/26), *IncN* (3.8%;1/26), *IncFIA* (3.8%;1/26), *IncP* (3.8%;1/26), *IncFrepB* (3.8%;1/26) (Table 8). Kalaycı-Yuksek et al., in their study conducted in our country in 2023, found that 28 strains containing both *bla*_NDM_ and *bla*_OXA-48_, *IncL*/M (7.7%) has determined [19].

In terms of *IncY*, it was 3.2% in Malaysia and 8.1% in Iran [20, 21]. The fact that *IncY* was found to be 30.7% in our study is an indication of the increased spread of *IncY* plasmids in our country. In a study conducted by Zhou et al. [22]. in China, they found *IncI1* to be 1.03%. In the study by Hosny et al. [23] in Egypt, *IncI1* was found to be 3.12%. In our study, *IncI1* was found to be 76.9%. Our results were found to be quite high compared to other studies.

When we compare with the studies conducted worldwide in terms of *IncFIA*, *IncFIB*, *IncN*, *IncP*, *IncFrepB*, and *IncL/M* types, our present results show that the rate of these types is lower or on the average of the data [20–24, 15]. In our study, *IncHI1* was found to be 3.8%. This is the first record in Türkiye and this may be an indication that *IncHI1* has just started to spread in our country. But in our study, *IncHI2*, *IncX*, *IncW*, *IncFIC*, *IncA*/*C*, *IncT*, *IncFIIs*, *IncK/B*, and *IncB/O* plasmid profiles were not found. We believe that this is due to regional differences and the fact that the spread has not yet occurred.

The fact that plasmids were detected in most of the strains suggests that resistance is an indicator of spread by plasmid. In this study, plasmid profiles that we investigated in *K. pneumoniae* in clinical samples for the first time in our country were examined, and the first data on the frequency of plasmids were presented. In particular, very high rates of *IncI1* and *IncY* plasmid types were detected. The presence of these plasmid profiles in our country brings with it the concern that antibiotic resistance is spreading rapidly and that these plasmid types will be more common in our country in the future. The increase in carbapenem resistance is largely driven by conjugative plasmids. Such plasmids carry multiple or single markers of resistance such as *bla*_NDM_ and *bla*_OXA-48_ [25].

This study provides an overview of the major plasmid families occurring in multiple antibiotic-resistant strains of *K. pneumoniae*. To our knowledge, this study represents the first report of *IncHI1* record in Türkiye. In addition, co-production of NDM-1 and OXA-48 carbapenemases in Türkiye was first performed by Kilic and Baysallar, it is clear that this resistance has increased in Türkiye in the last eight years [26]. OXA-48 was detected in all strains except strains 11 and 28. The fact that strains have at least one of the detected plasmid types shows the importance of the presence of plasmids in the rapid spread of OXA-48 and other resistance genes and also explains the increasing resistance rate and difficult treatment processes.

## Data Availability

No datasets were generated or analysed during the current study.

## References

[1] Meng X, Yang J, Duan J (2019). Assessing Molecular Epidemiology of Carbapenem-resistant *Klebsiella pneumoniae* (CR-KP) with MLST and MALDI-TOF in Central China. Sci Rep.

[2] Candan ED, Aksöz N (2015). *Klebsiella pneumoniae*: characteristics of carbapenem resistance and virulence factors. Acta Biochim Pol.

[3] Wayne PAC, and Laboratory Standars Institute. ClinicalLaboratory Standards Institute (CLSI) Performance standards for antimicrobial susceptibility testing. Document M100-S25: CLSI 2015. http://www.clsi.org/. Accessed 19 May 2023.

[4] Copur Cicek A, Saral A, Iraz M, Ceylan A, Duzgun AO, Peleg AY (2014). OXA and GES-type β-lactamases predominate in extensively drug-resistant *Acinetobacter baumannii* isolates from a Turkish University Hospital. Clin Microbiol Infect.

[5] Copur Cicek A, Saral A, Ozad Duzgun A, Yaşar E, Cizmeci Z, Balci PO (2013). Nationwide study of *Escherichia coli* producing extended-spectrum β-lactamases TEM, SHV and CTX-M in Turkey. J Antibiot.

[6] Poirel L, Heritier C, Tolün V, Nordmann P (2004). Emergence of oşacillinase mediated resistance to imipenem in *Klebsiella pneumoniae*. Antimicrob Agents Chemother.

[7] Carattoli A, Blertini A, Villa L, Falbo V, Hopkins KL, Threlfall EJ (2005). Identification of plasmids by PCR-based replicon typing. J Microbiol Methods.

[8] Delarampour A, Ghalehnoo ZR, Khademi F, Delarampour M, Vaez H (2019). Molecular detection of carbapenem-resistant genes in clinical isolates of *Klebsiella pneumoniae*. Ann Ig.

[9] Hazirolan G, Karagöz A (2020). Emergence of carbapenemase-producing and colistin resistant Klebsiella pneumoniae ST101 high-risk clone in Turkey. Acta Microbiol et Immunol Hungarica Vol.

[10] Central Asian and European Surveillance of Antimicrobial Resistance. Annual report 2020. https://www.who.int/europe/publications/i/item/WHO-EURO-2020-3469-43228-60585. Accessed 15 Novamber 2022.

[11] Poirel L, Bonnin RA, Nordmann P (2012). Genetic features of the widespread plasmid coding for the carbapenemase OXA-48. Antimicrob Agents Chemother.

[12] Ece G, Tunc E, Otlu B, Aslan D, Ece C (2018). Detection of *bla*OXA-48 and clonal relationship in carbapenem resistant *K. pneumoniae* isolates at a tertiary care center in Western Turkey. J Infect Public Health.

[13] Sağıroğlu P, Hasdemir U, Gelmez GA, Aksu B, Karatuna O, Söyletir G (2018). Performance of RESIST-3 O.K.N. K-SeT immunochromatographic assay for the detection of OXA-48 like, KPC, and NDM carbapenemases in *Klebsiella pneumoniae* in Turkey. Brazilian J Microbiol.

[14] Hawkey J, Wyres KL, Judd LM, Harshegyi T, Blakeway L, Wick RR (2022). ESBL plasmids in *Klebsiella pneumoniae*: diversity, transmission and contribution to infection burden in the hospital setting. Genome Med.

[15] Deniz Ögeday E, Cömert F, Köktürk F, Külah C, Aktaş E. Genişlemiş Spektrumlu Beta-Laktamaz (GSBL) Üreten *Escherichia coli* ve *Klebsiella spp*. İzolatlarında CTX-M Enzimlerinin Belirlenmesi. Türk Mikrobiyol Cemiyeti Dergisi. 2016;46(2):88–96.

[16] Iraz M, Düzgün AÖ, Sandallı C, Doymaz MZ, Akkoyunlu Y, Saral A (2015). Distribution of β-Lactamase genes among Carbapenem-resistant *Klebsiella pneumoniae* strains isolated from patients in Turkey. Ann Lab Med.

[17] Sahin K, Tekin A, Ozdas S, Akin D, Yapislar H, Dilek AR (2015). Evaluation of carbapenem resistance using phenotypic and genotypic techniques in *Enterobacteriaceae* isolates. Ann Clin Microbiol Antimicrob.

[18] Bektas A, Güdücüoğlu H, Gürsoy NC, Berktaş M, Gültepe BS, Parlak M (2018). Genişlemiş Spektrumlu Beta-Laktamaz (GSBL) Üreten *Escherichia coli* ve *Klebsiella pneumoniae* Suşlarının GSBL Genlerinin Araştırılması. Flora.

[19] Kalayci-Yuksek F, Gumus D, Uyanik-Ocal A, Gun G, Bayirli-Turan D, Macunluoglu AC et al. Carbapenem and Colistin Resistance, Integrons and Plasmid Replicon Types in Multi-Drug Resistant *Klebsiella* Strains Isolated in Turkey. Clin Lab. 2023 1;69(3). 10.7754/Clin.Lab.2022.220503 36912308

[20] Aslani S, Kiaei S, Afgar A, Morones-Ramırez JR, Aratboni HA, Faridi A et al. Determination of incompatibility group plasmids and copy number of the *bla*_NDM-1_ gene in carbapenem-resistant *Klebsiella pneumoniae* strains recovered from different hospitals in Kerman. Iran J Med Microbiol 2021;70(5). 10.1099/jmm.0.001361 33999798

[21] Al-Marzooq F, Mohd Yusof MY, Tay ST (2015). Molecular analysis of antibiotic resistance determinants and plasmids in Malaysian isolates of Multidrug resistant *Klebsiella pneumoniae*. PLoS ONE.

[22] Zhou H, Zhang K, Chen W, Chen J, Zheng J, Liu C (2020). Epidemiological characteristics of carbapenem-resistant Enterobacteriaceae collected from 17 hospitals in Nanjing district of China. Antimicrob Resist Infect Control.

[23] Hosny AEM, Rasmy SA, Aboul-Magd DS, Kashef MT, El-Bazza ZE (2019). The increasing threat of silver-resistance in clinical isolates from wounds and burns. Infect Drug Resist.

[24] Rodriguez-Navarro J, Miro E, Brown-Jaque M, Hurtado JC, Moreno A, Muniesa M (2020). Comparison of commensal and clinical isolates for diversity of plasmids in Escherichia coli and *Klebsiella pneumoniae*. Antimicrob Agents Chemother.

[25] Arabaghian H, Salloum T, Alousi S, Panossian B, Araj GF, Tokajian S (2019). Molecular characterization of Carbapenem resistant *Klebsiella pneumoniae* and *Klebsiella quasipneumoniae* isolated from Lebanon. Sci Rep.

[26] Kilic A, Baysallar M (2015). The first *Klebsiella pneumoniae* isolate co-producing OXA-48 and NDM-1 in Turkey. Ann Lab Med.

